# Advances and Applications of 3D Printing and Additive Manufacturing: Current Progress, Challenges, and Future Outlook

**DOI:** 10.3390/ma19173743

**Published:** 2026-09-03

**Authors:** Derui Jiang, Haopeng Shen, Aijun Huang

**Affiliations:** 1 Department of Paediatrics, The University of Melbourne, Melbourne, VIC 3052, Australia; 2 Murdoch Children’s Research Institute, Parkville, VIC 3052, Australia; 3 Commonwealth Scientific and Industrial Research Organisation, Melbourne, VIC 3168, Australia; haopeng.shen@outlook.com; 4 Department of Materials Science and Engineering, Monash University, Melbourne, VIC 3800, Australia; aijun.huang@monash.edu

## 1. Introduction

Additive manufacturing (AM) has evolved from a rapid-prototyping tool into a family of digitally driven manufacturing technologies capable of producing complex, lightweight, functionally graded, and highly customizable components. In metal AM, the layer-wise interaction between an energy source and feedstock enables near-net-shape production but also generates steep and spatially variable thermal cycles. These cycles govern melt-pool stability, solidification, microstructure, residual stress, distortion, defect formation, and ultimately component performance [1,2,3].

Consequently, the successful manufacture of a complex geometry does not, by itself, demonstrate that a component can be produced reproducibly or is fit for service. Anisotropy in additively manufactured Ti-6Al-4V [4], persistent ductility and fatigue challenges in steels [5], and microstructural and mechanical heterogeneity in Ni-based superalloys [6] illustrate the continuing need to connect process conditions with structure and properties.

At the same time, AM enables capabilities that cannot readily be achieved using conventional manufacturing, including architected lattices, flexible and multi-material devices, surface repair, customized implants, and digitally designed assistive technologies. These opportunities are shifting research from demonstrating manufacturing feasibility toward achieving application-specific functionality and translation. Against this background, the present Special Issue brings together six contributions spanning broad reviews of AM technologies and materials, flexible materials, surface safety, laser-cladded composite coatings, and biomedical applications. Collectively, these studies demonstrate the breadth of modern AM while emphasizing a common requirement: design freedom must be supported by quantitative understanding, reliable manufacturing, realistic performance assessment, and appropriate pathways toward standardization and adoption.

## 2. An Overview of the Published Articles

Additive manufacturing is moving decisively beyond rapid prototyping toward the production, repair, and customization of functional components. Recent developments include lightweight and bioinspired architectures, multi-material and flexible systems, directed-energy processes for surface engineering, and image-based workflows for patient-specific medical devices. These advances increasingly connect material selection, digital design, process control, and end-use performance. Nevertheless, several knowledge gaps continue to impede broader adoption. Robust material–process–structure–property relationships remain incomplete; defects, interfaces, and anisotropy are not yet consistently controlled; and performance is often assessed under simplified laboratory conditions rather than realistic combinations of load, wear, moisture, contamination, ageing, or biological exposure. Standardized qualification, scalable production, cost-effectiveness, and sustainability also remain important challenges. The six papers in this Special Issue address these issues across multiple material classes, manufacturing routes, and application environments while identifying priorities for future research.

Ramos et al. (contribution 1) provide a broad review of AM technologies, materials, and weight-optimization strategies, with particular attention to innovative printing patterns and bioinspired structures. By linking process families with metals, polymers, ceramics, composites, and smart materials, the review demonstrates how architecture can be treated as a design variable for improving strength-to-weight performance and reducing material use. It also identifies artificial intelligence, digital twins, robotics, hybrid manufacturing, and functionally graded materials as important drivers of the next generation of AM. Importantly, the authors emphasize that technological capability must be matched by repeatability, certification, environmental assessment, and economically viable scale-up. Future research should therefore move from isolated demonstrations toward integrated and traceable manufacturing systems in which generative design, process simulation, and quality assurance operate within a closed-loop framework.

Li et al. (contribution 2) narrow the focus to the rapidly growing field of 3D-printed flexible materials for soft robotics, wearable electronics, sensing, actuation, and biomedical applications. Their review covers material extrusion, vat photopolymerization, powder bed fusion, binder jetting, and hybrid approaches while highlighting persistent challenges including pore formation, weak interlayer bonding, limited surface quality, and long-term instability. A central gap is the absence of comprehensive correlations linking ink or feedstock characteristics, processing conditions, structural design, and functional response. Promising research directions include smart-responsive and biodegradable elastomers and hydrogels, stronger multi-material interfaces, multimodal printing platforms, and real-time defect correction through in situ monitoring and feedback control. Shared databases and standardized testing protocols for fatigue, durability, and biocompatibility will also be important if flexible devices are to progress from laboratory prototypes to reliable products.

Wieczorek et al. (contribution 3) investigate the slip resistance of fused-filament-fabricated PLA, PET-G, and TPU surfaces under dry and wet conditions. They show that material type and test environment significantly affect slip resistance, whereas printing direction and the measured roughness parameter do not exert statistically significant effects within the investigated range. TPU performs best under dry conditions and PLA under wet conditions, illustrating that a surface optimized for one environment may not remain optimal under another. This study opens a comparatively underexplored area of AM surface safety. Future work should consider a wider range of materials and textures, post-processing strategies, multiscale surface characterization, and testing that incorporates wear, contamination, and long-term use.

Hu et al. (contribution 4) examine IN718 and IN718-WC composite coatings deposited on EA4T steel by laser cladding, an important AM-enabled route for repair and surface enhancement. Increasing WC content raises microhardness and reduces wear volume, with the hardness at 15 wt.% WC reaching approximately 1.3 times that of the unreinforced IN718 coating; however, the lowest average coefficient of friction occurs at 10 wt.% WC. Tensile testing also reveals orientation-dependent behaviour associated with the cladding strategy and resulting microstructure. These findings underline the need for multi-objective optimization rather than maximization of a single property. Future studies should clarify WC dissolution, phase stability, particle distribution, interfacial bonding, and residual-stress development using advanced three-dimensional and in situ characterization. Linking these features to fatigue, corrosion-wear, crack initiation, and component-scale service performance would provide a stronger basis for qualified remanufacturing.

The two biomedical studies illustrate both the translational value of AM and the evidence still required before routine clinical implementation. Kaneko et al. (contribution 5) compare heat-cured and additively manufactured denture-base resins and show that ozonated water, ultrasonic cleaning, and ultraviolet irradiation each reduce adherent *Candida albicans*, while their combined application produces the greatest reduction in viable cells. The comparable response of the two fabrication routes is encouraging, although residual organisms remain on the surfaces and the study uses a single strain under in vitro conditions. Future investigations should examine mature multispecies biofilms, complex denture geometries and irradiation paths, repeated cleaning cycles, and the effects of ozone and ultraviolet exposure on resin chemistry, surface condition, and mechanical durability. Such data could support the development and clinical validation of automated, chemical-minimizing cleaning devices tailored to digitally manufactured dentures.

Trebacz et al. (contribution 6) integrate computed tomography, segmentation, CAD/CAM, finite element analysis, patient-specific guides, and metal AM to compare three Ti-6Al-4V stabilizers for canine atlantoaxial instability. All three constructs remain within the modelled material and biological safety limits, but they transfer load differently: the extended ventral plate distributes load across three vertebrae, whereas the dorsal construct reduces implant stress at the cost of higher bone stress. Cadaveric procedures further indicate shorter implantation times for dorsal fixation. This combination of computational and procedural evidence provides a valuable model for patient-specific device development. Future work should incorporate cyclic and multidirectional loading, experimental biomechanical validation, more realistic representations of bone quality and biological fixation, larger cohorts, and prospective clinical follow-up. In particular, the long-term consequences of bone-centred load transfer and the inability of the dorsal approach to facilitate arthrodesis require careful evaluation.

Taken together, the contributions to this Special Issue show that the future of AM lies not simply in increasing geometric complexity, but in demonstrating durable and application-relevant function. Progress will depend on integrating materials development, process control, predictive modelling, and validation rather than optimizing these elements independently.

## 3. Challenges and Future Outlook

From the authors’ view, a central challenge for the next stage of AM is to convert manufacturing feasibility into predictable and certifiable performance. For metal AM, alloy printability, thermal distortion, residual stress, lack-of-fusion defects, anisotropy, and microstructural heterogeneity remain interconnected rather than independent problems [1,2,3,4,5,6]. Progress will require experimentally validated multiphysics models, in situ monitoring of melt-pool and layer quality, and feedback-control strategies capable of identifying and correcting deviations during a build. These tools could increasingly be integrated within uncertainty-aware digital frameworks that predict not only nominal properties but also spatial variability and component service life. Standardized reporting of feedstock history, process parameters, post-processing, defect populations, and mechanical testing will remain essential for meaningful comparison across machines and laboratories.

An equally important quality challenge is the difference between intended CAD geometry and the structure that is actually manufactured. Recent work on electron-beam-melted Ti-6Al-4V simple cubic lattices illustrates this issue, showing that strut cross-sectional shape can influence dimensional fidelity, stress concentration, anisotropy, and compressive performance [7]. Such findings demonstrate that the behaviour of architected structures cannot always be predicted reliably from idealized geometry alone. Future studies should therefore increasingly integrate micro-CT-based inspection and as-built finite element models with geometric compensation and data-driven approaches to process optimization. The effects of build direction, surface-adhered particles, internal porosity, residual stress, and microstructure should also be separated systematically. Extending this approach to other lattice topologies and to multiaxial, fatigue, permeability, and biological testing would help establish stronger quality-control criteria for load-bearing applications.

Beyond metals, the increasing use of flexible, multi-material, and surface-engineered AM systems creates additional challenges in interface integrity, durability, environmental sensitivity, and long-term functional stability. The studies in this Special Issue demonstrate that performance can depend strongly on interactions among material selection, processing route, surface state, and service environment. Future qualification strategies should therefore move beyond isolated mechanical or surface measurements toward test protocols that reproduce realistic combinations of loading, wear, ageing, moisture, contamination, and other application-specific conditions. For multi-material and responsive systems, particular attention will be required to interfacial bonding, fatigue, functional degradation, and reproducibility across manufacturing platforms.

3D printing for biomedical applications represents a particularly compelling, albeit demanding, future direction. Rutz et al. [8] reported clinically meaningful long-term improvements in pain, functional scores, and hip coverage following 168 one-stage hip reconstructions in 121 children and young people with cerebral palsy. Three-dimensional CT was used to evaluate complex displacement and support operative positioning, providing a strong clinical basis for developing patient-specific 3D-printed anatomical models and surgical guides for pelvic and femoral osteotomies. Such guides could improve the transfer of planned correction angles, osteotomy locations, implant positions, and screw trajectories to the operating theatre. Before routine adoption, however, their accuracy, sterilization, operative efficiency, radiation implications, cost-effectiveness, and effect on complications and long-term outcomes must be established in prospective comparative studies. For non-operative management, Peng et al. [9] demonstrated how scanning, 3D printing, experimentation, and finite element analysis can be combined to tune ankle-foot orthosis stiffness through material, thickness, and trimline selection. Future orthoses should incorporate growth accommodation, objective prescription targets, fatigue and comfort testing, gait-based validation, and potentially embedded sensing for longitudinal monitoring. Implantable architected materials provide another promising direction. Additively manufactured auxetic metamaterials, for example, have been proposed for applications including hip stems, fixation devices, spinal cages, scaffolds, and stents [10]. Their tunable negative Poisson’s ratio may offer opportunities to modify bone–implant contact, reduce stress shielding, and improve fixation. Translation, however, will depend on reproducible fabrication, standardized fatigue testing, surface and biological characterization, and rigorous in vivo and clinical evaluation. More broadly, integrating surgical guides, adaptive orthoses, and implantable architected materials within a common image-to-design-to-manufacture workflow could enable increasingly personalized care, provided that engineers, clinicians, patients, manufacturers, and regulators are involved throughout development and validation.

AM research should also evaluate sustainability and affordability alongside technical performance. Energy and material consumption, powder or polymer reuse, post-processing, inspection burden, and end-of-life options should be assessed through life-cycle and techno-economic analysis. The field will advance most effectively through shared benchmark geometries and datasets, interoperable digital workflows, and prospective validation under realistic service conditions. By combining process science with quality assurance and application-led design, AM can progress from impressive demonstrations to dependable manufacturing solutions with measurable engineering and clinical benefit.
